# Kinetics of Macroion Adsorption on Silica: Complementary Theoretical and Experimental Investigations for Poly-l-arginine

**DOI:** 10.1021/acs.langmuir.4c03766

**Published:** 2025-01-21

**Authors:** Maria Morga, Dominik Kosior, Małgorzata Nattich-Rak, Izabella Leszczyńska, Piotr Batys, Zbigniew Adamczyk, Alexander M. Leshansky

**Affiliations:** † Jerzy Haber Institute of Catalysis and Surface Chemistry, Polish Academy of Sciences, Niezapominajek 8, PL30239 Krakow, Poland; ‡ Department of Chemical Engineering, Technion-IIT, Haifa 32000, Israel

## Abstract

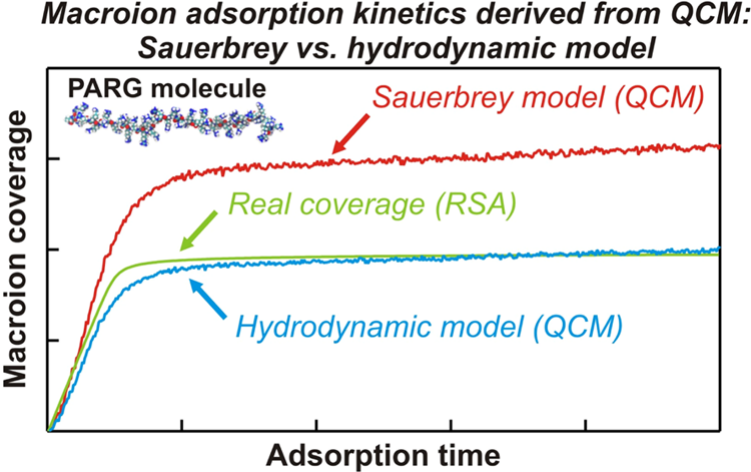

A comprehensive approach enabling a quantitative interpretation of poly-l-arginine (PARG) adsorption kinetics at solid/electrolyte interfaces was developed. The first step involved all-atom molecular dynamics (MD) modeling of physicochemical characteristics yielding PARG molecule conformations, its contour length, and the cross-section area. It was also shown that PARG molecules, even in concentrated electrolyte solutions (100 mM NaCl), assume a largely elongated shape with an aspect ratio of 36. Using the parameters derived from MD, the PARG adsorption kinetics at the silica/electrolyte interface was calculated using the random sequential adsorption approach. These predictions were validated by optical reflectometry measurements. It was confirmed that the molecules irreversibly adsorbed in the side-on orientation and their coverage agreed with the elongated shape of the PARG molecule predicted from the MD modeling. These theoretical and experimental results were used for the interpretation of the quartz crystal microbalance measurements carried out under various pH conditions. A comprehensive analysis unveiled that the results stemming from the hydrodynamic theory postulating a lubrication-like (soft) contact of the macroion molecules with the sensor adequately reflect the adsorption kinetics. The range of validity of the intuitively used Sauerbrey model was also estimated. It was argued that acquired results can be exploited to control macroion adsorption at solid/liquid interfaces. This is essential for the optimum preparation of their supporting layers used for bioparticle immobilization and shell formation at nanocapsules in targeted drug delivery.

## Introduction

Macroions are widely used in biotechnology and medicine for modification of substrates for protein and enzyme immobilization, separation, and biosensing processes.^1,2^ Especially, consecutive adsorption of cationic and anionic macroions according to the layer-by-layer (LBL) technique has been effectively applied in nanocapsule formulation for gene and DNA vaccines and drug delivery.^3−6^

Charged polymer macromolecules, comprising polypeptides, represent a particularly interesting group of macroions used in biomedical applications due to their biocompatibility and biodegradability.^2,3,6^ Additionally, they are stimuli-sensitive, enabling adjustment of their physicochemical properties by external conditions, such as temperature, pH, or ionic strength.^7^ In particular, the dependence of polypeptide molecule conformations on pH has been exploited in the design of materials for the controlled release of drugs in various therapies,^8^ hydrogels,^9^ and dressing materials.^10^

Because of its vital significance for practical applications, especially in the field of sensor construction,^11,12^ macroion adsorption on solid/electrolyte interfaces was extensively studied by various experimental techniques such as ellipsometry,^13,14^ reflectometry,^15,16^ surface plasmon resonance (SPR),^17^ infrared spectroscopy (IR),^18^ and electrokinetic methods.^19,20^ In comparison to these rather demanding methods, the quartz crystal microbalance (QCM) exhibits pronounced advantages, enabling sensitive, *in situ* measurements of adsorption/desorption kinetics under flow conditions for a broad range of molecule sizes. Therefore, this technique has been extensively applied to investigate the formation of the macroion layers used as support for the immobilization of bioparticles such as DNA,^21^ proteins,^22^ viruses,^23^ and bacteria.^24^ However, despite the wide range of applications, macroion adsorption at solid/electrolyte interfaces is mostly studied qualitatively and remains insufficiently understood.

In ref (25), the influence of pH on poly-l-arginine (PARG) and poly-l-lysine (PLL) adsorption on a silica surface was investigated using QCM and the streaming potential methods. It was shown that at pH up to 9, the macroion layers exhibited sufficient stability, whereas at higher pH values, they were characterized by greater coverage but limited stability.

Porus et al.^26,27^ studied the adsorption of PLL at a silica surface over a wide range of NaCl concentrations (10^–3^–10^–1^ M) using optical reflectometry and QCM. Comparing the QCM coverage calculated from the Sauerbrey equation with that obtained from reflectometry, the layer thickness and water content in adsorbed PLL layers were determined. It was shown that the layer thickness monotonically increased with the ionic strength, which was interpreted as an indication of a side-on adsorption mechanism of the macroion.

Barrantes et al.^28^ investigated the formation of PLL/heparin (HEP) multilayers adsorbed under various pH values on silica and gold substrates using QCM and ellipsometry. Using the viscoelastic model to interpret the QCM data, it was postulated that, under acidic conditions, a side-on conformation of PLL molecules prevailed, whereas at pH 8.5, the molecules adopted α-helical conformation.

It should be mentioned that a quantitative interpretation of the experimental results pertinent to macroion adsorption at solid substrates was not attempted due to the lack of essential information about the molecule size, their conformations, and the electrokinetic charge under various physicochemical conditions. Additionally, in the case of QCM measurements, the interpretation was hampered by the lack of an adequate theoretical model, yielding sensor impedance for various overtones. As a result, despite extensive experimental efforts, the basic mechanisms of macroion adsorption at charged surfaces still remain inadequately understood.

Therefore, to acquire valid information about macroion adsorption kinetics under flow conditions, a comprehensive approach, combining theoretical modeling with thorough experimental measurements, was developed in this work. Poly-l-arginine (PARG), which exhibits pronounced biocompatible properties and is widely used in drug and gene delivery systems,^29^ for the preparation of wound healing dressings,^30^ in biosensing,^31^ and in cancer immunotherapy.^32^

Applying the all-atom molecular dynamics (MD) modeling, the basic physicochemical parameters of the PARG molecule were determined. These data, primarily comprising the chain diameter, the contour length, and the radius of gyration, enabled effective modeling of PARG adsorption in terms of the hybrid random sequential adsorption (RSA)—convective-diffusion approaches. The theoretical results were validated by optical reflectometry measurements, enabling a thorough analysis of the QCM measurements in terms of various models comprising the commonly used Sauerbrey model and the recently proposed hydrodynamic model. It was shown that the latter provides a quantitative interpretation of kinetic data derived from the QCM, yielding precise information about the real macroion coverage.

It is worth mentioning that the obtained results are of practical interest because the QCM measurements have also been used for humidity control,^33^ alcohol classification,^34^ in brewery,^35^ and in the food industry for fouling investigation.^36^

## Materials and Methods

### Materials

PARG, used in this work, was purchased from Sigma-Aldrich (Merck KGaA, Germany). The average molar mass, determined by dynamic viscosity measurements in previous work,^37^ was equal to 42 kg mol^–1^.

Ultrapure water was obtained from the Milli-Q Elix & Simplicity 185 purification system (Merck Millipore, USA). The NaCl, HCl, and NaOH were supplied by Sigma-Aldrich (Merck KGaA, Germany).

The macroion solutions were directly prepared before each experiment, dissolving an appropriate amount of dried crystalline powder in NaCl solutions of a given ionic strength and pH adjusted using HCl and NaOH solutions.

### Methods

The diffusion coefficient of the PARG molecule was determined by the dynamic light scattering technique using the Zetasizer Nano ZS instrument (Malvern Panalytical, United Kingdom). The hydrodynamic diameter was calculated by using the Stokes–Einstein relationship. The electrophoretic mobility of molecules was measured by the laser Doppler velocimetry technique using the same apparatus. The zeta potential was calculated from the Ohshima equation,^38^ pertinent to anisotropic (cylindrical) molecules.

On the other hand, the zeta potential of the QCM-D sensor was acquired via the streaming current measurements using the SurPASS (Anton Paar GmbH, Austria). Two sensors, cleaned directly before each experiment using a diluted piranha solution, ultrapure water, and a UV–ozone cleaner, were mounted into the instrument on both sides of the flow cell. The streaming current *I* was measured as a function of applied hydrostatic pressure difference Δ*p*. The zeta potential, ζi, is calculated from the Helmholtz–Smoluchowski relationship^39^
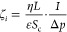
1where η is the dynamic viscosity of the electrolyte, *L* is the length of the channel, *S*_c_ is its cross-section area, and ε is the electric permittivity of the electrolyte. The measurement precision was improved by measuring the streaming current under various pressure differences.

The adsorption kinetics of PARG on Si/SiO_2_ wafers (Silchem, Germany) was investigated using *in situ* optical reflectometry within a microfluidic impinging—jet cell, as previously described.^40,41^ The solid substrate was carefully cleaned before each experiment using piranha solution (H_2_SO_4_/H_2_O_2_ 1:1) and ultrapure water. The fixed-angle reflectometer was equipped with a polarized green diode laser working at a wavelength of 532 nm (World Star Tech TECGL–532 Series, Canada). The reflectometer cell consisted of a capped equilateral dispersing prism made out of quartz with a borehole of *r*_b_ = 0.5 mm radius and a spacer providing a gap of *h*_b_ = 0.85 mm between the surface and the prism. The reflected light was divided using a beam splitter into the perpendicular and parallel components, which were acquired with two photodiodes. The dry mass of the adsorbates was calculated from the reflectometry signal using a homogeneous slab model.^40,42^ The macroion solution of a bulk concentration of 1 mg L^–1^ was flushed through the cell with a regulated volumetric flow rate, typically 1.66 × 10^–3^ cm^3^ s^–1^.

The QCM measurements were carried out using a Q-Sense QCM Instrument (Biolin Scientific, Sweden). Quartz sensors with a fundamental frequency of 5 MHz were supplied by Biolin Scientific. Before each experiment, the sensor was cleaned according to the procedure described by Reinhardt and Kern.^43^ Briefly, a mixture of 96% sulfuric acid (H_2_SO_4_), hydrogen peroxide (30%), and ultrapure water in the volume ratio 1:1:1 was prepared, and the sensor was immersed in the solution for 2 min. Afterward, the sensor was rinsed with ultrapure water and boiled in ultrapure water at 80 °C for 30 min. Finally, the sensor was dried in a nitrogen gas stream. The silica substrate is characterized by low roughness and defined electrokinetic charge; thus, it is frequently used as a model substrate for the investigation of the nanospecies adsorption phenomenon.

Before each experiment, a stable baseline for a pure electrolyte of ionic strengths equal to 100 mM NaCl and a given pH was obtained. Afterward, the PARG suspension of a fixed mass concentration was flushed through the cell at a fixed flow rate set to 1.33 × 10^–3^ cm^3^ s^–1^. After a plateau value was attained, the desorption run was initiated by flushing through the cell a pure electrolyte solution of the same ionic strength and pH in order to determine the stability of the formed macroion layers.

The topography of the QCM sensors was determined by AFM imaging carried out under ambient air conditions in a semi-contact mode using the silicon probes and polysilicon cantilevers HA–NC Etalon with resonance frequencies of 140 kHz ± 10% or 235 kHz ± 10% and the NT–MDT Olympus IX71 device with the SMENA scanning head.^44^ Main topographical parameters comprising the root-mean-square (rms), the surface height, the skewness characterizing the height distribution asymmetry, and the roughness correlation length are provided in the Supporting Information. All measurements were performed at 25 °C (298 K).

## Theoretical Modeling

### MD Modeling

The GROMACS 2022.3 package was used for all-atom MD modeling of the fully charged poly-l-arginine (PARG) molecule consisting of 50 monomers (repeat units).^45,46^ The molecule structure was generated using Avogadro software.^47^ The Amber force field, specifically the ff99SB-ILDN, was applied to describe PARG and ions,^48^ while the TIP3P model was employed for water.^49^ It should be mentioned that overbinding of ions is a common and well-known problem in classical molecular dynamics simulations due to the absence of electronic polarizability in the force fields.^50^ This results in conformations and dynamics of macromolecules being dependent on the force field choice.^51^ The PARG molecule was solvated, and the Na^+^ and Cl^–^ ions were added to set the ionic strength equal to 100 mM and neutralize the system. The simulation box size was 14.9 × 14.9 × 14.9 nm^3^, with the periodic boundary conditions applied in all directions. Energy minimization was then performed, followed by a 150 ns long production run in the NPT ensemble. First, 50 ns was considered as an equilibration and was disregarded from the analysis. The V-rescale thermostat and the isotropic Parrinello–Rahman barostat were used to control the temperature and pressure.^52,53^ The temperature was set to 298 K and the pressure to 1 bar, with the coupling constants 0.1 and 2 ps, respectively. In order to apply the 2 fs time-step, all of the bonds in the PARG molecule and water molecules were controlled by the LINCS and SETTLE algorithms, respectively.^54,55^ The long-range electrostatic interactions were calculated using the PME method.^56^ The VMD software package was used for the visualizations.^57^ Gromacs built-in functions, *gmx polystat* and *gmx rdf*, were used to extract end-to-end (EtE) distance and radius of gyration, as well as to calculate radial distribution function (RDF).

### Hybrid Convective Diffusion—RSA Modeling

The kinetic runs derived from QCM measurements were interpreted using the theoretical data derived from the hybrid approach, where the bulk transport was described by a phenomenological convective–diffusion equation coupled with the surface boundary layer transport equation where the fluid convection effects were neglected.^58^ The mass transfer rates for the QCM cell appearing in the surface transport equation were previously obtained in AFM calibration experiments.^59^ The available surface function (also referred to as the surface blocking function), reflecting the probability of macroion adsorption, was calculated from the formula derived by applying the scaled particle theory.^60^ The maximum macroion coverage at various pH values was calculated using the equivalent hard particle approach. A detailed description of these calculations is presented in the Supporting Information.

## Results and Discussion

### Theoretical Modeling Results

**Figure 1:**
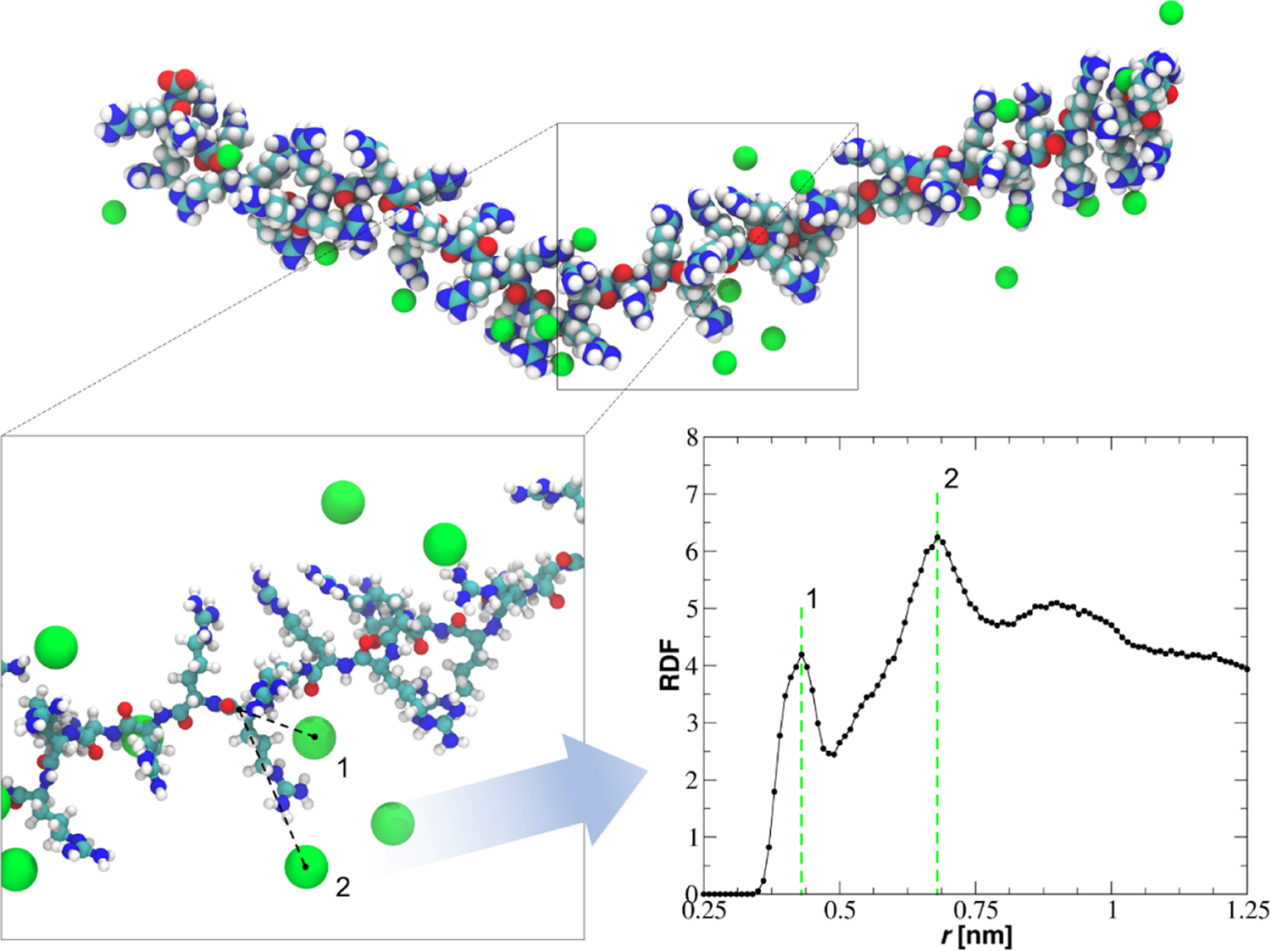
On top there is shown the snapshot of the PARG molecule containing 50 repeat units derived from MD simulations at 100 mM NaCl. All atoms are shown as spheres of radius equal to their van der Waals radius. The N, O, C, H, and Cl atoms are colored in blue, red, cyan, white, and green, respectively. On the bottom, the zoom of the chain fragment is shown with the marked position of the Cl^–^ counterions condensing at different distances from the PARG backbone. On the right, the RDF function between Cα and Cl^–^ counterions is shown. Vertical dashed lines correspond to the peak’s maxima.

In order to characterize the basic properties of the PARG moleculein 100 mM NaCl, extensive MD modeling was performed. This enabled the acquisition of information about molecule conformations, characterized in terms of the end-to-end (EtE) distance fluctuations in time with its average value, the radius of gyration, and the equivalent chain diameter, bare and with condensed counterions. In Figure 1, a snapshot of PARG molecule conformation at 100 mM NaCl is shown, along with the RDF between Cα and Cl^–^ counterions. As can be readily seen, despite a relatively large salt concentration (close to the physiological conditions), the molecule adopts a rather extended conformation. This is in contrast to significant counterion condensation, which is often predicted for other strong macroions.^61^ A closer inspection of the counterion position reveals that they can occupy space between the side groups or can be located outside the molecule. This was quantitatively confirmed via the RDF function, which exhibits two peaks: (1) located at the distance *r* ∼ 0.43 nm, corresponding to the Cl^–^ ions located between the side chains, and (2) located at *r* ∼ 0.69 nm, corresponding to Cl^–^ ions forming an outer layer of condensed counterions. The position of the second peak allows to predict that the effective diameter of the PARG molecule with condensed counterions is equal to 1.4 nm.

**Figure 2:**
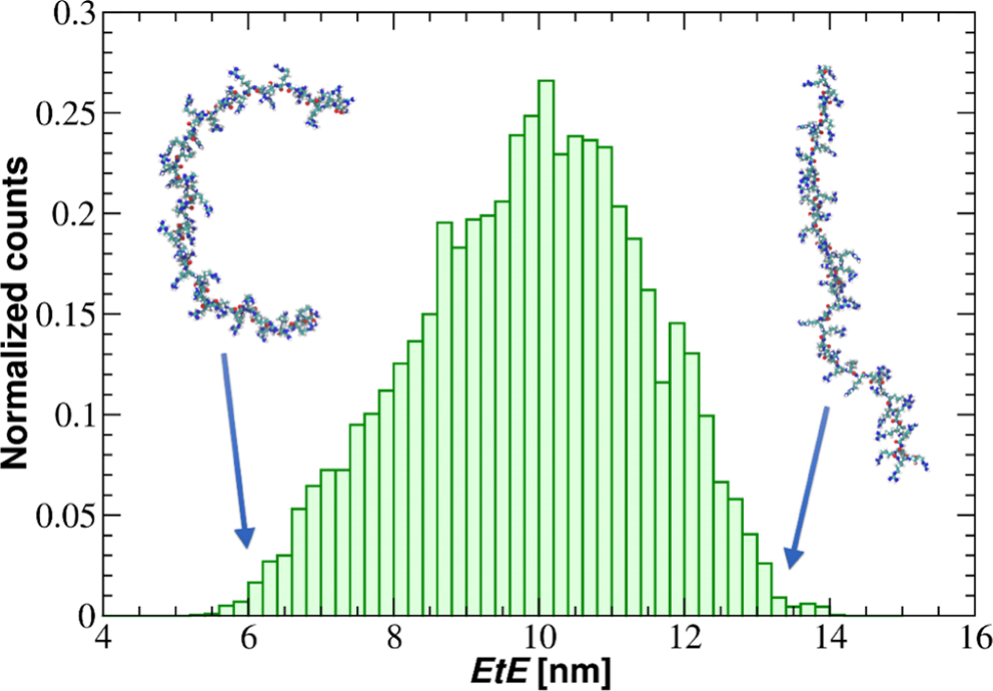
EtE distance distribution for the PARG molecule consisting of 50 monomers derived from MD modeling in 100 mM NaCl. The insets correspond to the compacted and extended molecule conformation.

In Figure 2, the EtE distribution of the PARG molecule, in the form of a histogram, is shown. The Gaussian-like distribution of EtE suggests that the PARG molecule can adopt both compacted and extended shapes. The average EtE distance and radius of gyration are 9.88 ± 0.02 and 3.50 ± 0.01 nm, respectively.

A linear relationship was applied to estimate the end-to-end distance of the polymer chains. According to the classical scaling theory in polymer physics, the scaling relationship between the EtE distance or radius of gyration (*R*_g_) and the degree of polymerization (*N*) should be less than 1 for a random coil conformation. A scaling exponent of 1 applies only to fully extended chains. However, considering the aspect ratio of 36, corresponding to an elongated shape, the scaling exponent approaches 1. This indicates that the elongated shape dominates PARG conformations, including the EtE distance and the *R*_g_.

It was also confirmed in the modeling that the basic parameters, such as the monomer length and the bare and the effective chain diameter with condensed counterions, were independent of the size of the molecules (the number of monomers) ranging from 20 to 50. Therefore, these basic parameters were used to calculate the properties of the larger PARG molecule used in the experiments, whose molar mass was equal to 42 kg mol^–1^ (Table 1). It consisted of 240 monomers, given that the molar mass of the monomer is equal to 0.174 kg mol^–1^. Using this value and the monomer contour length of 0.2 nm derived from modeling, one obtains 48 nm as the extrapolated molecule contour length. Similarly, the estimated EtE distance and radius of gyration were equal to 47.4 and 16.8 nm, respectively.

**Table 1:** Primary and Derivative Parameters of PARG Molecule Determined by MD Modeling for 100 mM NaCl, pH 5.8–7.4, *T* = 298 K^a^

quantity (unit), symbol	value	remarks
average molar mass [kg mol^–1^], *M_n_*	42 ± 2	viscosity method^37^
monomer molar mas [kg mol^–1^], *M*_1_	0.174	from chemical composition
density [kg m^–3^], ρ_p_	1.5 ± 0.04 × 10^3^	modeling^37^
average number of monomers in the molecule, *N*_m_	240	calculated as *M*_*n*_/*M*_1_
molecule volume [nm^3^], *ν*_p_	47	calculated as 10^27^ × *M*_*n*_/(ρ_p_*N*_Av_)
equivalent sphere diameter [nm]	4.5	calculated as (6*v*_p_/π)^1/3^
monomer length [nm], *l*_m_	0.20 ± 0.02	modeling
bare chain diameter [nm], *d*_b_	1.1 ± 0.02	modeling
chain diameter with condensed counterions [nm], *d*_c_	1.4 ± 0.1	modeling
contour length [nm]	48	modeling/extrapolation
equivalent cylinder contour length [nm], *L*_e_	50	calculated as
average aspect ratio parameter, λ	36	calculated from the equivalent cylinder, *L*_e_/*d*_c_

a *N*_Av_—Avogadro number.

Additionally, using the molar mass and the density of the molecule, ρ_p_ = 1.5 g cm^–3^, the molecule volume is calculated to be 47 nm^3^. Therefore, using the equivalent chain diameter with condensed counterions of 1.4 nm derived from MD modeling, one can calculate the equivalent cylinder contour length to be equal to 50 nm, which agrees with the contour length of the molecule calculated by extrapolation. For the sake of convenience, all of the primary parameters derived from MD modeling and the extrapolated derivative parameters are collected in Table 1.

### Bulk and Surface Characteristics of the Substrates

To properly interpret adsorption kinetic measurements, the bulk characteristics of PARG molecules, including the electrophoretic mobility, the zeta potential, and the diffusion coefficient, were acquired using the above-described ELS and DLS methods. Analogously, the zeta potential of the QCM sensor was acquired by streaming current measurements.

**Figure 3:**
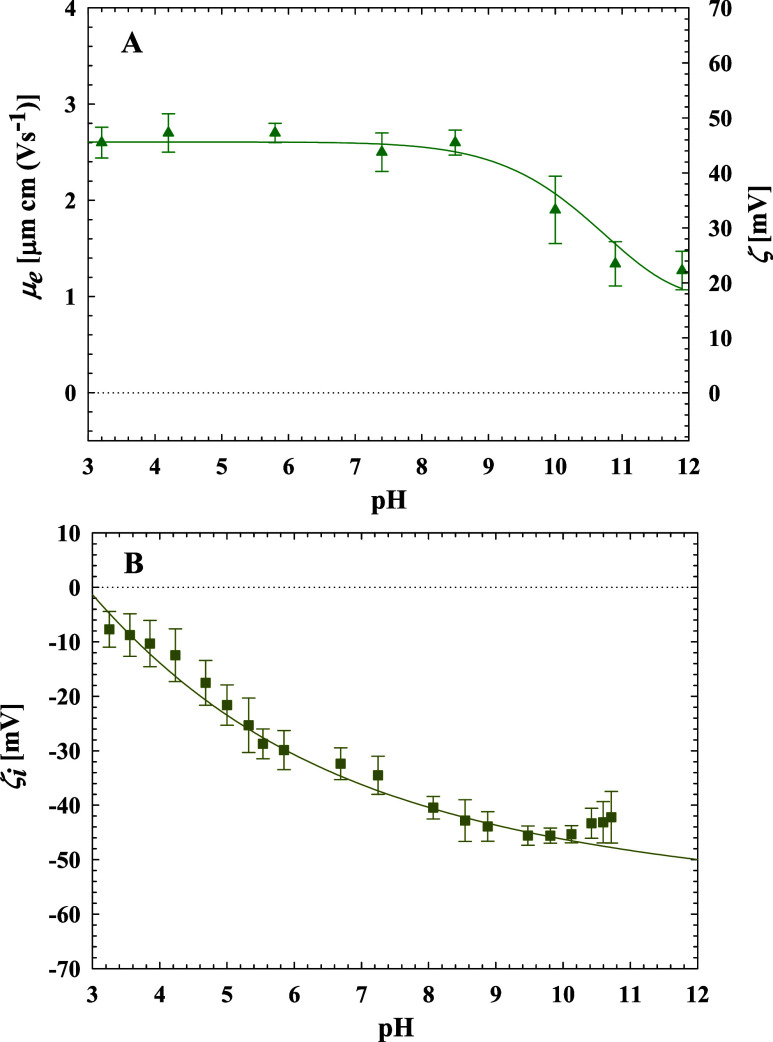
(A) Dependence of the electrophoretic mobility, μ_e_ (left vertical axis), and the zeta potential, ζ (right vertical axis) of PARG molecules on pH in 100 mM NaCl. (B) The dependence of the zeta potential of the silica sensor, ζ_*i*_ on pH in 100 mM NaCl. The solid lines are guides to the eye.

The measurement results are shown in Figure 3, illustrating the dependence of the electrophoretic mobility (μ_e_) and the zeta potential of PARG molecules (ζ) on pH (Figure 3A), and the zeta potential of the silica sensor (ζ_*i*_) on pH (Figure 3B). As seen, the electrophoretic mobility of PARG molecules was positive across the entire pH range, attaining the largest value of 2.7 μm cm (V s^–1^) at pH 3.2, which practically remained constant up to pH 9.0. Above pH 9.0, a slight decrease in the electrophoretic mobility was observed, down to 1.9 μm cm (V s^–1^) at pH 10.2. These mobility data correspond to zeta potentials of 49 (±3) and 36 (±3) mV at pH 3.2 and 10.2, respectively. In contrast, the zeta potential of the silica sensor was negative for the entire range of pH, varying between −10 and −50 mV for pH of 3.2 and 10.8, respectively, as shown in Figure 3B.

The diffusion coefficient of PARG molecules directly measured by DLS varied between 4.5 (±0.3) × 10^–7^ and 5.4 (±0.3) × 10^–7^ cm^2^ s^–1^ for pH 4.0 and 10.2. This corresponds to the hydrodynamic diameter change (calculated from the Stokes-Einstein equation) from 11 to 9 nm, respectively. Using the electrophoretic mobility and the diffusion coefficient data, the electrokinetic charge of the PARG molecule *q*_e_ was calculated from the Lorentz–Stokes formula
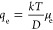
2where *k* is the Boltzmann constant and *T* is the absolute temperature.

Equation 2 applies to arbitrary charge distribution and molecular shapes. However, its accuracy decreases if the electric double-layer thickness becomes comparable with the molecule chain diameter. The number of elementary charges per molecule can be calculated as *N*_c_ = *q*_e_/*e*, where *e* is the elementary charge equal to 1.602 × 10^–19^ Coulombs.

The physicochemical parameters, such as the diffusion coefficients, the hydrodynamic diameters, the electrophoretic mobility, the zeta potentials, and the number of uncompensated charges per molecule calculated from eq 2, are collected in Table 2. It is noteworthy that the zeta potentials of the PARG molecule and the sensor have opposite signs across the entire pH range, which facilitates effective adsorption of the PARG molecules onto the sensor.

**Table 2:** Physicochemical Parameters of the PARG Molecule Determined at Various pH Values for 100 mM NaCl, *T* = 298 K^a^

pH	*D* (×10^–7^) [cm^2^ s^–1^]	*d*_H_ [nm]	μ_e_ [μm cm (V s^–1^)]	ζ [mV]	ζ_I_ [mV]	*N*_c_
4.0 ± 0.2	4.-5 ± 0.3	11 ± 2	2.7 ± 0.2	49 ± 2	–12 ± 5	15
5.7 ± 0.3	4.9 ± 0.3	10 ± 2	2.7 ± 0.2	49 ± 3	–30 ± 4	14
7.4 ± 0.3	4.9 ± 0.3	10 ± 2	2.5 ± 0.2	42 ± 3	–34 ± 4	13
10.2 ± 0.2	-5.2 ± 0.4	9.0 ± 3	1.9 ± 0.2	36 ± 2	–43 ± 3	9.0

a *D*: diffusion coefficient, *d*_H_: hydrodynamic diameter, μ_e_: electrophoretic mobility, ζ: bulk zeta potential of PARG molecule, ζ_*i*_: zeta potential of silica substrate, and *N*_c_: number of uncompensated charges per molecule.

### RSA Calculations of PARG Adsorption Kinetics

The modeling of PARG adsorption kinetics on the silica sensor was carried out by applying the hybrid RSA approach. The mass transfer rates were calculated using the diffusion coefficient determined by DLS and the available surface function was calculated from the scaled particle theory using the physicochemical molecule characteristics derived from the MD modeling.

This enabled the determination of the entire adsorption kinetic runs comprising the maximum PARG coverage at various pHs for a side-on adsorption mechanism (a detailed description is presented in the Supporting Information). The hybrid RSA approach assumes that adsorption is irreversible within the examined adsorption time and that molecules are randomly adsorbed upon contact with available sites until the maximum coverage is reached. Physically, this corresponds to the situation when the species are irreversibly bound to the sites due to short-range attractive interactions of an electrostatic or chemical nature. Furthermore, PARG adsorption was assumed to be irreversible and localized, which means that the molecule position at the site remained fixed during the entire simulation run.^60^

The dimensionless maximum coverages Θ_mx_ slightly increased with pH from 0.35 to 0.43 mg m^–2^ at pH 4.0 and 10.2, respectively (Table 3). This effect was caused by the decrease in the electrostatic repulsion among adsorbing PARG molecules, whose zeta potential decreased from 49 to 36 mV and the pH varied between 4.0 and 10.2 (see Table 2). For comparison with experimental measurements, the maximum mass coverage Γ_mx_, expressed in mg m^–2^, was also calculated from the constitutive dependence
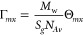
3where *S*_g_ is the PARG molecule cross-section area.

**Table 3:** Maximum Coverages of PARG on the Silica Sensor (Θ_mx_, Γ_mx_) Determined from the soft–RSA Modeling (Side-On Adsorption), Reflectometry, and from QCM-D (Calculated Using the Sauerbrey Equation) for Various pH values, 100 mM NaCl, and *T* = 298 K^a^

pH	RSA Θ_mx_ [1]	RSA Γ_mx_ [mg m^–2^]	reflectometry Γ_mx_ [mg m^–2^]	QCM-D Γ_Q_ [mg m^–2^]
4.0 ± 0.2	0.35 ± 0.02	0.45 ± 0.02	0.18 ± 0.02	0.22 ± 0.02 (*f*_3_)
		0.36 ± 0.02*		0.20 ± 0.02 (*f*_11_)
5.7 ± 0.3	0.37 ± 0.01	0.47 ± 0.02	0.37 ± 0.02	0.60 ± 0.03 (*f*_3_)
		0.38 ± 0.02*		0.55 ± 0.03 (*f*_11_)
7.4 ± 0.3	0.40 ± 0.01	0.52 ± 0.02	0.45 ± 0.02	0.56 ± 0.04 (*f*_3_)
		0.43 ± 0.02*		0.53 ± 0.04 (*f*_11_)
10.2 ± 0.2	0.43 ± 0.02	0.56 ± 0.03	0.82 ± 0.04	1.00 ± 0.05 (*f*_3_)
		0.45 ± 0.02*		0.95 ± 0.05 (*f*_11_)

a Molar mass 42 kg mol^–1^; density 1.5 g cm^–3^, cross-section area for bare molecule 53 nm^2^ (MD), *cross-section area for molecule with condensed counterions 67 nm^2^ (MD).

Considering the data derived from MD modeling (Table 1), one can estimate *S*_g_ to be equal to 53 and 67 nm^2^ for the bare molecule (chain diameter of 1.1 nm) and the molecule with condensed counterions (chain diameter 1.4 nm), respectively. Using the latter value of the cross-section area, the predicted maximum mass coverage of PARG was equal to 0.38 and 0.43 mg m^–2^ at pH 4.0 and 10.2, respectively (Table 3).

**Figure 4:**
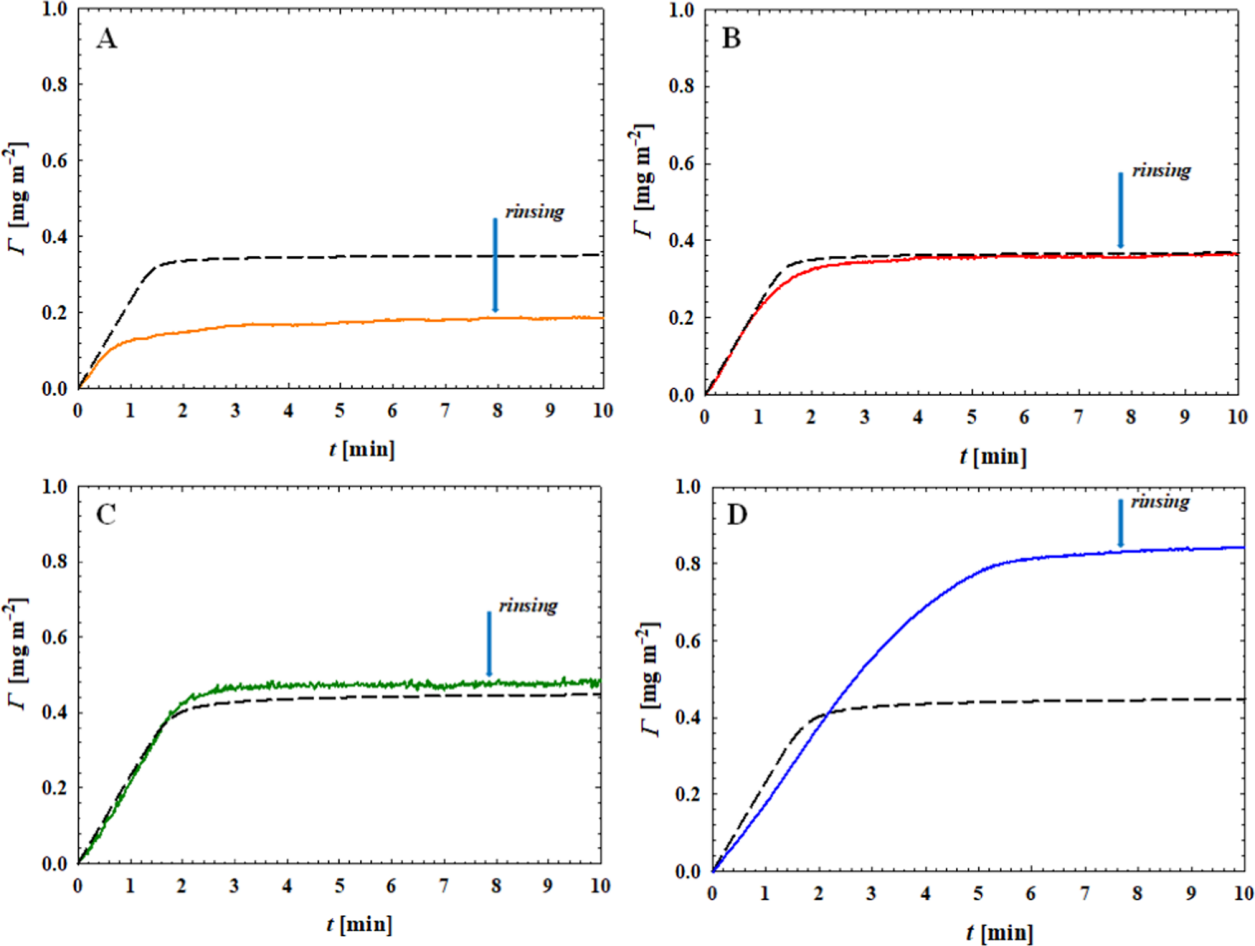
Kinetics of PARG molecules adsorption on silica derived from reflectometry measurements at various pH values: (A) pH 4.0; (B) pH 5.7; (C) pH 7.4; and (D) pH 10.2. All measurements were performed for 100 mM NaCl, bulk macroion concentration 1 mg L^–1^, volumetric flow rate *Q* = 1.66 × 10^–3^ cm^3^ s^–1^ and expressed as the dependence of the mass coverage (Γ) on the adsorption time. The dashed lines denote the theoretical results derived from the hybrid RSA model, and the arrows show the beginning of the desorption run.

To validate the theoretical results derived from RSA modeling, reflectometric measurements were performed, in which the kinetics of PARG adsorption onto silicon/silica plates was investigated at various pH values. The results of these experiments are depicted in Figure 4.

The adsorption kinetic runs shown in Figure 4 yielded the maximum PARG coverages of 0.37 and 0.45 mg m^–2^ at pH 5.7 and 7.4, respectively, which match (within experimental error bounds) the RSA predictions (Table 3). However, the maximum coverage at pH 4.0, equal to 0.18 mg m^–2^, was significantly lower than the RSA coverage of 0.35 mg m^–2^. This effect was attributed to the insufficient adhesion strength of PARG molecules controlled by electrostatic interactions because the zeta potential of silica was equal to −13 mV at pH 4.0 compared to −30 mV at pH 5.7. In consequence, only weakly bound particles could be removed by the hydrodynamic shearing forces induced by the electrolyte flow. It should be mentioned that this effect was not considered in the RSA modeling. On the other hand, at pH 10.2, the initial adsorption kinetics of PARG was markedly slower compared to pH 5.7 and 7.4 (see Supporting Information), and the maximum coverage attained 0.82 mg m^–2^, which exceeds almost two times the RSA coverage of 0.45 mg m^–2^. This behavior, quantitatively analyzed previously,^62^ can be attributed to the slow aggregation of PARG molecules at this pH, which results in the increase in their molar mass compared to single molecules. Considering eq 3, one can predict that mostly dimeric PARG molecules are formed at a pH of 10.2.

These results indicate that the optimum pH range, where single-molecule PARG layers of controlled coverage are formed on silica, is between pH 5.0 and 8.0.

### QCM-D Measurements of PARG Adsorption

Compared to reflectometry, the QCM exhibits pronounced advantages, enabling *in situ* kinetic measurements for a plethora of sensor surfaces and adsorbates ranging from small macroion molecules to large polymer microparticles.^63^ However, a deconvolution of the QCM signals, in order to obtain the macroion mass coverage, requires an adequate theoretical model, more rigorous than the Sauerbrey equation, formally applicable for measurements of rigid adsorbed films in vacuum or gas phase and yet commonly used in the literature.^64,65^ Therefore, in this work, the experimental results derived from QCM measurements were also interpreted, except for the Sauerbrey model,^66^ in terms of the recently proposed hydrodynamic models.^67−69^

It should be mentioned that primary QCM signals are the normalized frequency −Δ*f*/*n*_o_ and the dissipation shifts Δ*D* recorded as a function of the adsorption time for various overtones (*n*_o_). Such dependencies acquired for various pH values and *I* = 100 mM NaCl are presented in the Supporting Information. Using the frequency shift signals for various overtones, the adsorbate coverage can be calculated from the general dependence
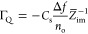
4where *C*_s_ = *Z*_q_/2*f*_0_^2^ is the Sauerbrey constant equal to 0.177 (mg m^–2^) Hz^-1^ for the fundamental frequency *f*_0_ of 5 × 10^6^ Hz, *Z*_q_ is the shear impedance of quartz, and *Z̅*_im_^–1^ is the imaginary component of the complex sensor impedance normalized by the pure inertia component.

**Figure 5:**
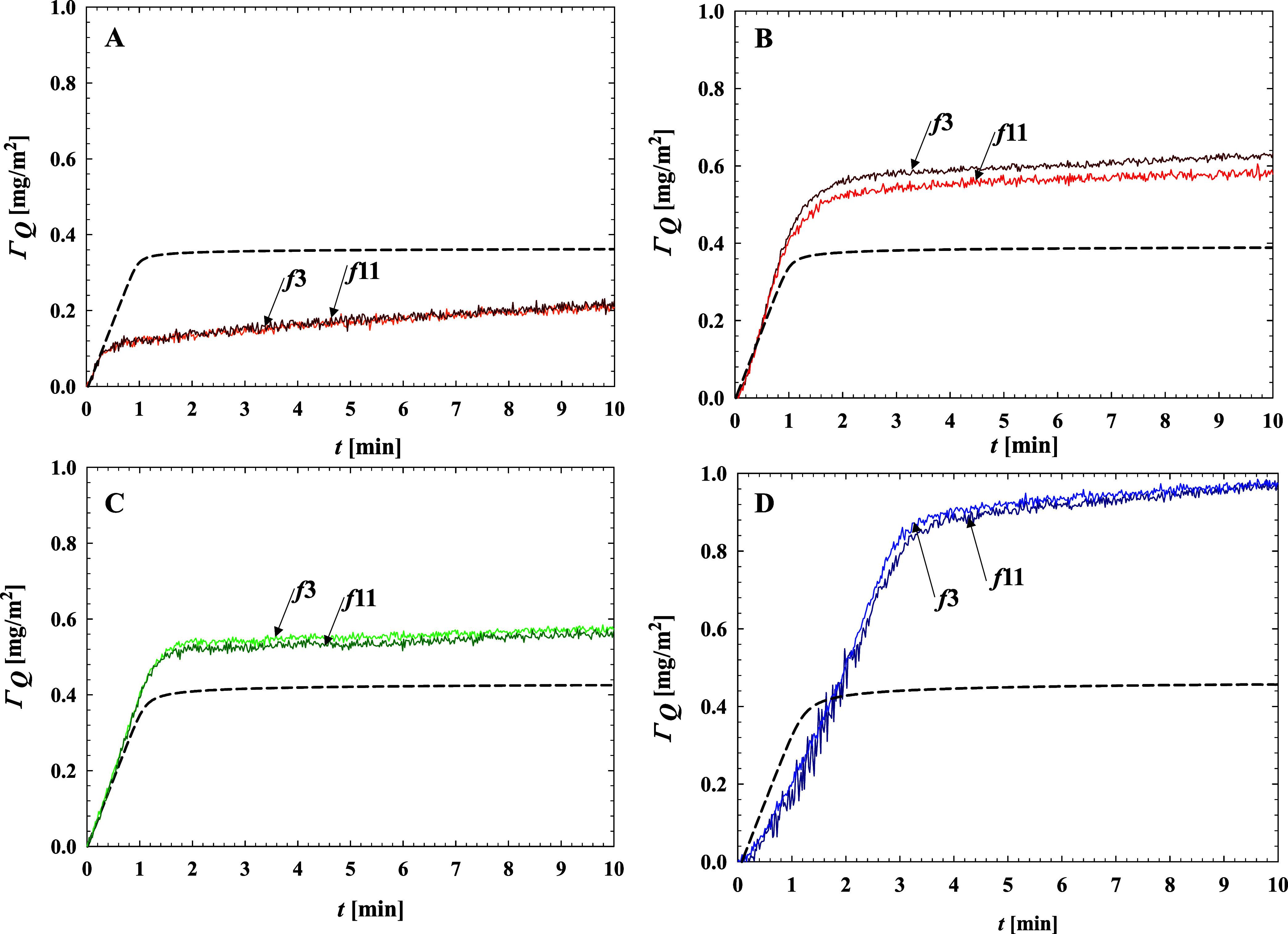
Kinetics of PARG molecules adsorption on silica sensor, at 100 mM NaCl and various pH values: (A) pH 4.0; (B) pH 5.7; (C) pH 7.4; and (D) pH 10.2, expressed as the dependence of the QCM–D coverage (Γ_Q_) calculated using the Sauerbrey equation on the adsorption time (for the 3rd and 11th overtones). Bulk macroion concentration is 5 mg L^–1^ and the volumetric flow rate equals 1.33 × 10^–3^ cm^3^ s^-1^.

In a general case, the impedance is a complicated function of the adsorbate size and shape, frequency, hydrodynamic boundary layer thickness, and the adsorbate coverage. In consequence, the relationship expressed by eq 4 becomes implicit and requires numerical inversion to calculate the coverage. Therefore, one often simplifies the analysis of QCM measurements, assuming that *Z̅*_im_^–1^ is equal to unity, independently of the coverage and any other parameters. This assumption physically corresponds to pure inertia (mass) load, where all hydrodynamic and specific forces are entirely neglected. In this case, the adsorption kinetics can be calculated from the simple formula, usually referred to as the Sauerbrey equation
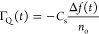
5A comparison of the PARG adsorption kinetics derived using eq 5 with that predicted from RSA modeling at various pH values is shown in Figure 5.

One can see that the QCM kinetic runs derived from the Sauerbrey model are qualitatively similar to those derived from the RSA modeling, particularly at pH 5.7 and 7.4, although the maximum coverages are significantly larger. At pH 5.8, they were equal to 0.60 and 0.55 mg m^–2^ for the third and the 11th overtone, respectively. At pH 7.4, the QCM coverage was equal to 0.56 and 0.53 mg m^–2^ for the third and the 11th overtone, respectively. Hence, the maximum coverage is by a factor of 1.3–1.5 times larger than this predicted from RSA modeling and reflectometric measurements. Considering eq 4, it indicates that the sensor impedance was also 30-50 % larger than that predicted from the Sauerbrey model.

The QCM–D data acquired at pH 5.8 and 7.4 were also interpreted in terms of the theoretical results derived by applying the *ab initio* type hydrodynamic theory.^67−69^ Although, in a general case, the impedances can only be calculated numerically, useful analytical solutions were derived for small values of the *d*_p_/2δ parameter, where *d*_p_ is the characteristic dimension of the adsorbate (for spherical particles *d*_p_ is equal to its diameter), δ = (2*v*/ω)^1/2^ = (*v*/π*n*_o_*f*_0_)^1/2^ is the hydrodynamic boundary layer thickness and *v* is the fluid kinematic viscosity. For the PARG molecule where *d*_p_ = 1.4 nm, the *d*_p_/2δ parameter was equal to 0.0051 and 0.0098 for the third and 11th overtones, respectively. Thus, for such low values of *d*_p_/2δ, the normalized impedance can be calculated from the following formula (Supporting Information)
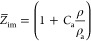
6where *C*_a_ is the dimensionless constant equal to 9.64 for spherical adsorbates and ρ and ρ_a_ are the fluid and the adsorbate densities, respectively.

On the other hand, for the soft contact of adsorbate molecules enabling their motion relative to the sensor surface, the imaginary impedance component can be calculated as (Supporting Information)
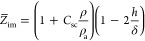
7where *C*_sc_ is the dimensionless constant, *h* = 2*a* + *h*_m_ is the distance between the adsorbate and the sensor, and *h*_m_ is the minimum distance between the adsorbate and the sensor surfaces.

Therefore, under the soft contact regime, using eq 4, the real macroion coverage can be explicitly calculated from the following formula
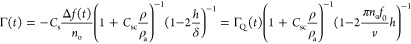
8It should be mentioned that these equations are strictly applicable for spherically shaped adsorbates in the limit of low surface coverage, where the *C*_sc_ constant was theoretically predicted to be 1.5.^69^ Unfortunately, at the present time, exact numerical calculations of this constant for elongated molecules are impractical. However, considering that this constant represents the ratio of the hydrodynamic to the inertia forces, one can exploit the results discussed in ref (70), where the elongated adsorbate was modeled as a sting of touching beads. It was shown that the ratio of the normalized hydrodynamic force for the string to a spherical adsorbate of the same coverage varied between 0.57 and 0.55 for a number of beads equal to 20 and 40, respectively. Considering that the aspect ratio for the PARG molecule was equal to 36, the value of the normalized force was assumed to be 0.56. Hence, *C*_sc_ = 0.84 (1.5 × 0.56) was used in the calculation presented hereafter.

Using the density of PARG given in Table 1 and considering that the minimum distance between the molecule and the silica surface is considerably smaller than the hydrodynamic boundary layer thickness for all overtones, one can transform eq 8 to a useful form
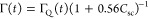
9This equation indicates that the PARG adsorption kinetics expressed in terms of a real (dry) coverage is proportional for all overtones to the kinetics derived from the Sauerbrey model given by eq 5.

**Figure 6:**
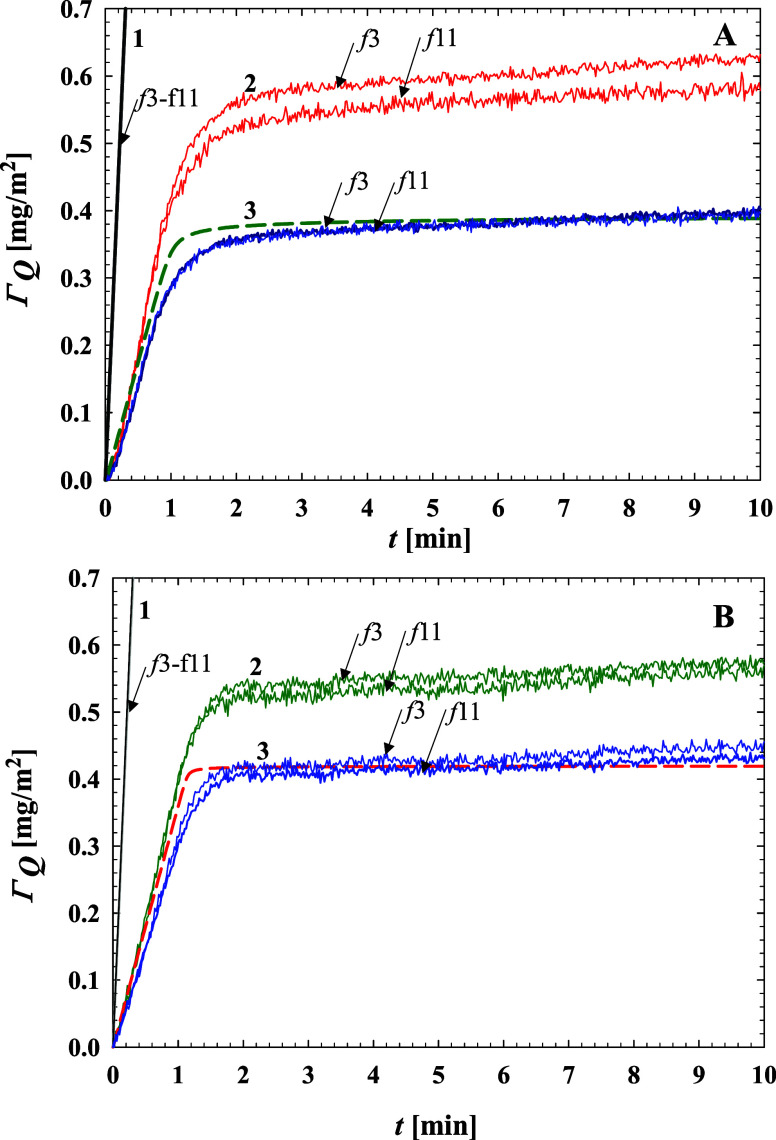
Comparison of the PARG adsorption kinetics on the silica sensor derived from QCM using various theoretical models with the RSA coverage: (A) pH 5.7: 1. Stiff contact hydrodynamic model; 2. Sauerbrey model; and 3. Lubricated (soft) contact hydrodynamic model. The dashed line shows the RSA coverage pertinent to pure inertia. (B) pH 7.4: 1. Stiff contact hydrodynamic model; 2. Sauerbrey model; 3. Lubricated (soft) contact hydrodynamic model. The dashed line shows the RSA coverage pertinent to pure inertia.

A comparison of the PARG adsorption kinetics derived from QCM measurements using various theoretical models with that acquired from the RSA model, which yields the real mass coverage, is shown in Figure 6 for pH 5.7 and 7.4.

The theoretical data pertinent to the stiff and soft contact hydrodynamic models were obtained using the PARG molecule density of 1.5 g cm^–3^ and the characteristic dimension of 1.4 nm equal to the chain diameter derived from MD calculations (Table 1). These results indicate that the lubricated (soft) contact hydrodynamic model can be applied for an adequate analysis of the QCM frequency change signals, enabling a quantitative prediction of PARG molecule adsorption kinetics on a silica sensor.

## Conclusions

A comprehensive approach enabling a quantitative interpretation of PARG adsorption kinetics at solid/electrolyte interfaces was developed. The first step involved all-atom MD modeling of physicochemical characteristics, yielding PARG molecule conformations, its contour length, and the cross-section area. It was also shown that PARG molecules, even in concentrated electrolyte solutions (100 mM NaCl), assume an elongated shape with an aspect ratio of 36.

Using the parameters derived from MD, the PARG adsorption kinetics at the silica/electrolyte interface were calculated using the random sequential adsorption approach. These predictions were validated by the optical reflectometry measurements. It was confirmed that the molecules were irreversibly adsorbed in the side-on orientation, and their coverage agreed with the elongated shape of the PARG molecule predicted from the MD modeling.

These theoretical and experimental results were used for the interpretation of the quartz crystal microbalance (QCM) measurements carried out at various pH values. A comprehensive analysis showed that the results stemming from the hydrodynamic theory postulating a lubrication-like (soft) contact of the macroion molecules with the sensor adequately reflect the adsorption kinetics. The range of validity of the intuitively used Sauerbrey model was also estimated.

It was argued that the acquired results could be exploited to control macroion adsorption at solid/liquid interfaces. This is essential for the optimal preparation of their supporting layers used for bioparticle immobilization and shell formation at nanocapsules in targeted drug delivery.

## Supporting Information Available

AFM characteristics of QCM sensor; modeling macroion adsorption/desorption kinetics—the hybrid RSA approach; and interpretation of the quartz microbalance results (PDF)
