# Supplementary material for: Kinetics of Macroion Adsorption on Silica: Complementary Theoretical and Experimental Investigations for Poly-l-arginine
Source: Langmuir. 2025 Jan 21;41(4):2248–58. doi: 10.1021/acs.langmuir.4c03766 (PMC11803736; doi:10.1021/acs.langmuir.4c03766)
Supplement: Supplementary file 1 — la4c03766_si_001.pdf [file la4c03766_si_001.pdf]

SUPPORTING INFORMATION

Kinetics of Macroion Adsorption on Silica: Complementary Theoretical and Experimental Investigations for Poly-L-Arginine

Maria Morga[1], Dominik Kosior[1], Małgorzata Nattich-Rak[1], Izabella Leszczyńska[1], Piotr Batys[1], Zbigniew Adamczyk[1]*, Alexander M. Leshansky [2],*

[1]Jerzy Haber Institute of Catalysis and Surface Chemistry, Polish Academy of Sciences, Niezapominajek 8, PL30239 Krakow, Poland.

[2] Department of Chemical Engineering, Teknion-IIT, Haifa 32000, Israel

*Corresponding authors:
Maria Morga, – maria.morga@ikifp.edu.pl
Zbigniew Adamczyk – zbigniew.adamczyk@ikifp.edu.pl

Number of pages: 12
Number of figures: 2
Number of schemes: 0
Number of tables: 1

Table of contents:

1. AFM Characteristics of QCM Sensor
2. Modeling Macroion Adsorption/Desorption Kinetics-the Hybrid RSA Approach
3. Interpretation of the Quartz Microbalance Results

1. AFM Characteristics of QCM Sensor

The topography of the sensors used in QCM measurements was determined by atomic force microscopy (AFM) imaging carried out under ambient conditions in a semi–contact mode. A typical image of the gold/silica sensor with the two surface height profiles are shown in Figure S1.

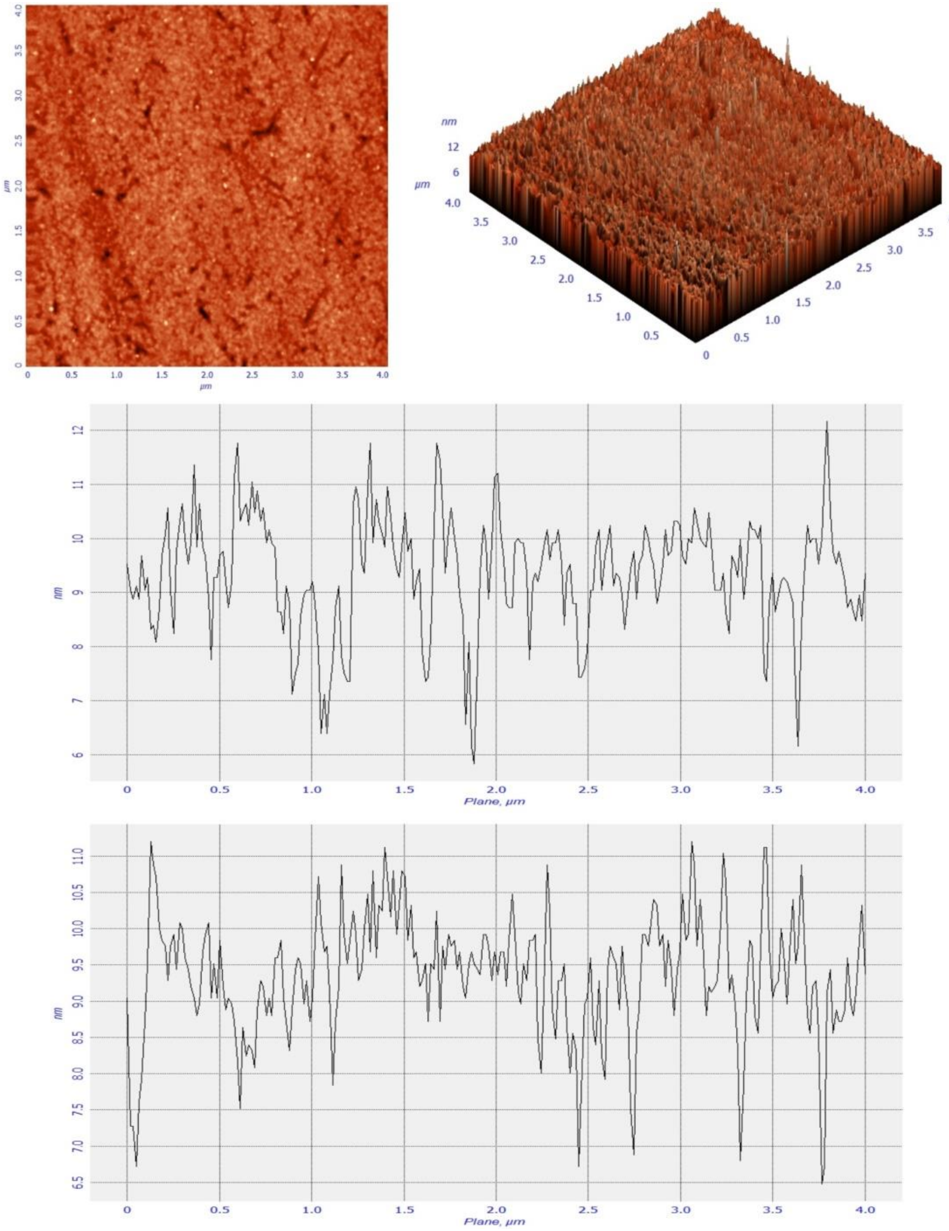

Fig. S1. AFM image of the gold/silica QCM sensor with two surface height profiles.

The average height ($\bar{h}$), the root mean square (*rms*) and the skewness (*sk*) of the sensor were calculated as follows:[1]

$$\bar{h} = \frac{1}{S}\int_s \left(h(\mathbf{r}_s) - h_0\right) d\mathbf{r}_s$$

$$rms^2 = \frac{1}{S}\int_s \left(h(\mathbf{r}_s) - \bar{h}\right)^2 d\mathbf{r}_s = \frac{1}{S}\int_s h^2(\mathbf{r}_s)\, d\mathbf{r}_s - \bar{h}^2 \qquad \text{(S1)}$$

$$sk = \frac{1}{rms^3 S}\int_s \left(h(\mathbf{r}_s) - \bar{h}\right)^3 d\mathbf{r}_s$$

where S is the projection area of the surface, $h(\mathbf{r}_s)$ is the local height of the surface profile measured relatively to the reference plane located at h_0, $\mathbf{r}_s$ is the surface position vector, and $\bar{h}$ the average height of a rough surface.

For a discrete set of surface heights h_i obtained from AFM scanning, the above parameters can be approximated by the following sums:

$$\bar{h} = \frac{1}{N_i}\sum_{i=1}^{N_i}\left(h_i - h_0\right)$$

$$rms^2 = \frac{1}{N_i}\sum_{i=1}^{N_i}\left(h_i - \bar{h}\right)^2 \qquad \text{(S2)}$$

$$sk = \frac{1}{rms^3 N_i}\sum_{i=1}^{N_i}\left(h_i - \bar{h}\right)^3$$

where N_i is the number of mesh points.

It is to remember, however, that the parameters calculated from Eq. (S2) depend on the size of the scanned area and the number of mesh points. Additionally, tip convolution effects may appear if the lateral dimension of the surface roughness becomes comparable with the AFM tip radius of curvature, typically of the order of 10 nm.[1]

Table S1. Basic topographical parameters of the gold/silica QCM sensors derived from the AFM measurements.

**Sensor**	**rms [nm]**	**Surface height [nm]**	**Skewness**	**Roughness correlation length [nm]**	**Roughness wavelength [nm]**
Silica	0.9 ± 0.1	2.5 ± 0.3	–0.70 ± 0.1	70±20	120 ± 20

These topographical parameters comprising the root mean square (*rms*), the surface height, the skewness characterizing the asymmetry of the height distribution, the roughness correlation length and the roughness wavelength are collected in Table S1.

It is interesting to underline that the roughness correlation length and wavelength of the silica sensor are almost two orders of magnitude larger than the *rms* parameter. This means that the sensor surface can be treated as very smooth from the point of view of adsorbates whose lateral dimension is smaller than 100 nm, such as the PARG macroion molecules.

2. Modeling Macroion Adsorption/Desorption Kinetics-the Hybrid RSA Approach

Adsorption kinetics under convective–diffusion transport conditions at solid substrates can be theoretically described using a hybrid approach exploiting the convective–diffusion equation:[2,3]

$$\frac{\partial n_a}{\partial t} = D\,\nabla^2 n_a - \frac{D}{kT}\nabla\cdot\left(\mathbf{F}\,n_a\right) - \mathbf{V}\cdot\nabla n_a \tag{S3}$$

where n_a is the number concentration of adsorbate particles (molecules) in the suspension (solution), t is the time, D is their translational diffusion coefficient, k is the Boltzmann constant, T is the absolute temperature, $\mathbf{F}$ is the external force vector and $\mathbf{V}$ is the unperturbed (macroscopic) fluid velocity vector.

Eq. (S3) is coupled with the surface layer transport equation where the fluid convection effects are negligible:

$$j_a = \frac{dN}{dt} = k_a\,n_a(\delta_a)\;B(N) - k_d N = k_c\;\frac{k_a}{k_a - k_c}[n_b - n_a(\delta_a)] \tag{S4}$$

where j_a is the net adsorption/desorption flux, N is the adsorbate surface concentration, k_a, k_d are the adsorption and desorption constants, $n_a(\delta_a)$ is the number concentration of adsorbate particles at the adsorption boundary layer of the thickness δ_a, $B(N)$ is the available surface (blocking) function, k_c is the bulk transfer rate constant and n_b is the number concentration of adsorbate particles in the bulk.

It should be mentioned that the adsorption and desorption constant can be calculated in an *ab initio* way if the particle/interface interaction potential is known.[2,3]

Under convective transport, where the adsorbate concentration $n_a(\delta_a)$ remains in a local equilibrium with the surface coverage, the constitutive expression for the adsorption flux, becomes:

$$j_a = \frac{K\,B(N) - K_d N}{(K-1)B(N)+1}k_c n_b \tag{S5}$$

where $K = k_a / k_c$ is the dimensionless coupling constants, $K_d = k_d / \left(S_g k_c n_b\right)$ is the dimensionless desorption constant, S_g is the characteristic cross-section area of the adsorbate particle.

In the case of laminar flows, such as for example the radial impinging jet flows, the mass transfer rate constants can be analytically calculated from the formula:[3]

$$k_c = C_f Q^{1/3} D^{2/3} \tag{S6}$$

where C_f is the parameter depending on the flow Reynolds number and the cell geometry, Q is the volumetric flow rate of the suspension and D is the solute diffusion coefficient.

Eqs. (S4)–(S5) can be expressed in the form of the definite integral:

$$\int_0^{\Gamma} \frac{\left(k_a - k_c\right) B(\xi) + k_c}{k_a c_b B\left(\xi\right) - k_d \xi} d\xi = k_c t \tag{S7}$$

where $\Gamma = N\, m_a$ is the mass coverage of the adsorbate layer (referred to as the dry coverage) m_a is the mass of a single particle, ξ is the dummy integration variable and $c_b = m_a n_b$ is the mass concentration of adsorbate particles in the bulk.

Eq. (S7) represents a general solution for particle deposition kinetics under convection driven transport. For bulk transport-controlled regime characterized by the condition $k_a >> k_c$ and a lower coverage range, Eq. (S7) simplifies to the linear form:

$$\Gamma = k_c c_b t \tag{S8}$$

It is worth underlining that this linear dependence of Γ on the time is universally valid for the low coverages under any convection-diffusion driven deposition regime, in particular under the flow conditions prevailing in the QCM cell.

However, to explicitly calculate particle deposition kinetics from Eq. (S7) under arbitrary coverage range the available surface function should be known. It can be conveniently acquired from the random sequential adsorption (RSA) modeling introducing the dimensionless (absolute) particle coverage Θ defined as:[4–7]

$$\Theta = N / S_g = \Gamma / (m_a S_g) \tag{S9}$$

In the case of anisotropic (elongated) adsorbates the available surface function for the side-on adsorption regime is given by the following expression:[3]

$$B\left(\Theta\right) = \left(1 - \Theta\right) e^{-\left(1+2\gamma_p\right)\frac{\Theta}{1-\Theta} - \gamma_p \left(\frac{\Theta}{1-\Theta}\right)^2} \tag{S10}$$

where the adsorbate particle shape parameter, γ_p, is defined as:

$$\gamma_p = \frac{P_r^2}{4\pi S_g} \tag{S11}$$

and P_r is the perimeter of the particle cross-section.

For larger coverage range a more adequate expression for the available surface was used:[3]

$$B(\Theta) = \left(1-\Theta^*\right) e^{-\left(1+2\gamma_p\right)\frac{\Theta^*}{1-\Theta^*} - \gamma_p\left(\frac{\Theta^*}{1-\Theta^*}\right)^2} \left(\frac{\Theta_{mx}-\Theta}{\Theta_{mx}-\Theta^*}\right)^4 \tag{S12}$$

where Θ^* is a transition coverage that can be chosen quite arbitrarily below the maximum coverage Θ_{mx}.

The maximum coverage of macroion molecules approximated by a string of touching beads can be calculated applying the coarse–grained random sequential adsorption (RSA) approach.[8] These results were generalized in Ref. [9] to cylindrical shape of molecules, where the following expressions was derived for rigid and flexible molecules, respectively:

$$\Theta_\infty = 0.80\frac{4}{\pi}(d_c / L_c)^{0.19} \qquad \text{rigid macroion molecules}$$

$$\Theta_\infty = 0.93\frac{4}{\pi}(d_c / L_c)^{0.17} \qquad \text{flexible macroion molecules} \tag{S13}$$

where Θ_∞ is the jamming coverage pertinent to non–interacting (hard) molecules, d_c and L_c are the molecule chain diameter and the contour length, respectively.

It was also shown in Ref. [3] that the above results can be extended to the case of adsorbate molecules interacting via the short-range repulsive Yukawa potential. For electrostatic double-layer interactions the characteristic range of this potential h^* is given by:

$$h^* = \frac{1}{\kappa d_c}\left[\ln\frac{\phi_o}{\phi_{ch}} - \ln\left(1+\frac{1}{\kappa d_c}\ln\frac{\phi_o}{\phi_{ch}}\right)\right] \tag{S14}$$

where $\kappa^{-1} = \left(\frac{\varepsilon k T}{2e^2 I}\right)^{1/2}$ is the electrical double–layer thickness, ε is the permittivity of the medium, e is the elementary charge, I is the ionic strength of the electrolyte solution, ϕ_o is electrostatic energy at contact and ϕ_{ch} is the characteristic interaction energy.[3]

Consequently, one can calculate the jamming coverage for interacting particles referred to as the maximum coverage) from the relationship:

$$\Theta_{mx} = \Theta_\infty \frac{1}{(1+h^*)^2} \tag{S15}$$

Knowing Θ_{mx} one can use Eq. (S12) to calculate the available surface function.

3. Interpretation of the Quartz Microbalance Results

It should be mentioned that primary signals derived from the quartz microbalance (QCM) measurements are the frequency shifts $-\Delta f(t)$ and the dissipation shifts $\Delta D(t)$ acquired as a function of the adsorption time for various overtones n_o.[10] For the sake of convenience the frequency shifts are often expressed in the normalized form $-\Delta f / n_o$ and the dissipation shifts are converted to the bandwidth shifts $\frac{1}{2} f_0 \Delta D(t) = \overline{\Delta D}$, where f_0 is the fundamental frequency of the sensor equal to 5×10^6 Hz. These parameters acquired from QCM measurements of PARG adsorption on gold/silica sensor at various pHs are presented in Fig. S2.

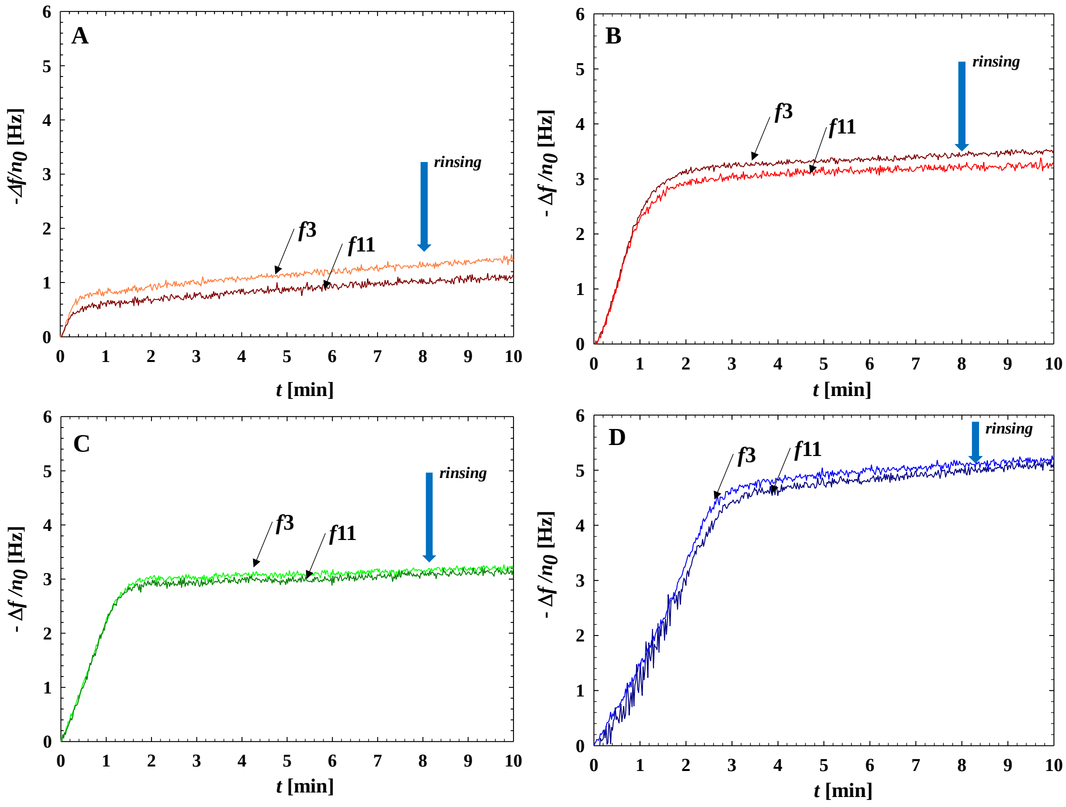

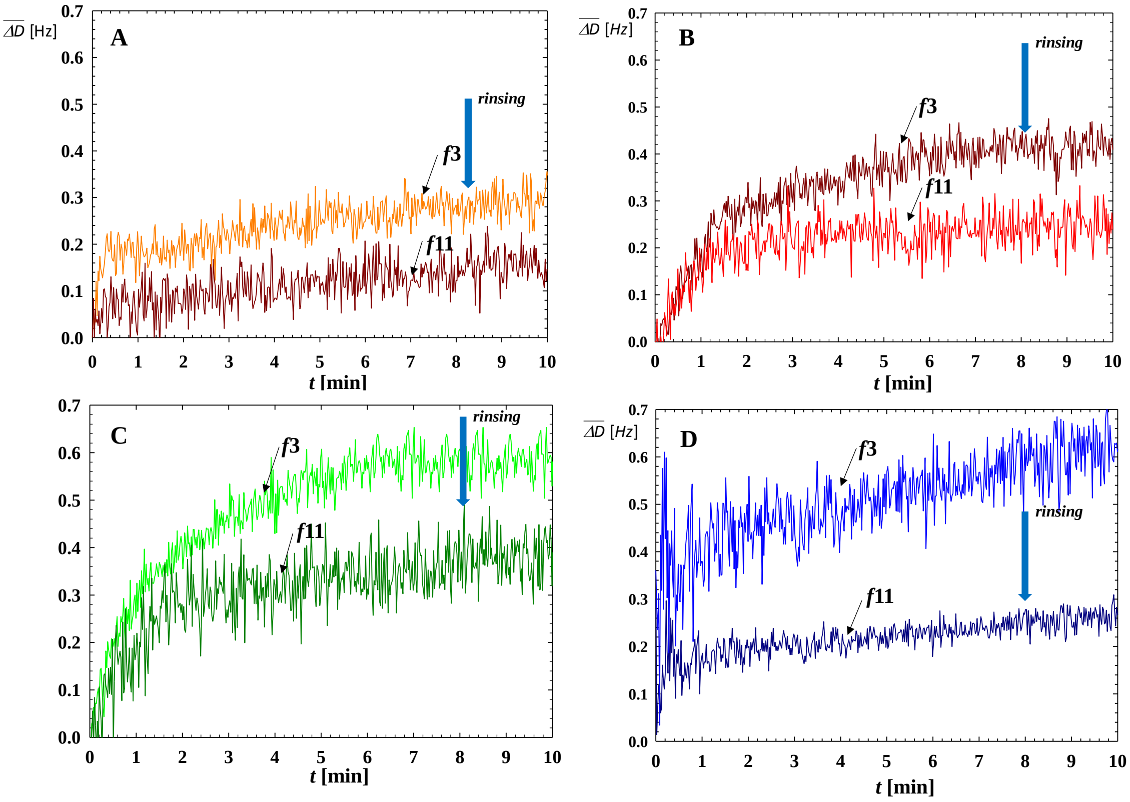

Fig. S2. The normalized frequency shifts $-\Delta f / n_o$ (expressed in Hz) and the bandwidth shifts $\overline{\Delta D}$ (in Hz) acquired from QCM measurements for PARG at various pHs: **(A)** pH 4.0; **(B)** pH 5.7; **(C)** pH 7.4; **(D)** pH 10.2 and on silica sensor for bulk macroion concentration of 5 mg L^{-1}, 100 mM NaCl, volumetric flow rate 1.33×10^{-3} cm^3 s^{-1}. The arrows show the starting time point of the desorption run where pure NaCl solution of proper pH was flushed through the cell.

Fig. S2 confirms that the frequency shifts were little dependent of the overtone number (3 to 11) and were characterized by an adequate precision showing low noise level. In contrast, the dissipation signals showed a considerable noise that can limit their use for the calculations of the complex sensor impedance ΔZ^* whose components are defined by the constitutive dependencies:[11–13]

$$\mathrm{Im}\left(\Delta Z^*\right) = Z_{im} = \frac{\pi Z_q n_o}{f_0} \, (-\Delta f / n_o)$$

$$\mathrm{Re}\left(\Delta Z^*\right) = Z_{re} = -\frac{\pi Z_q}{f_o} \overline{\Delta D} \qquad \text{(S16)}$$

where Z_q is the acoustic impedance of quartz equal to 8.8×10^6 kg m^{-2} s^{-1}.

For a purely inertia load the impedance components are given by:

$$\mathrm{Im}\left(\Delta Z^*\right) = 2\pi f_0 n_o \Gamma(t)$$

$$\mathrm{Re}\left(\Delta Z^{*}\right) = 0 \tag{S17}$$

where $2\pi f_0 n_o = \omega$ is the angular velocity of the sensor oscillations, f_0 is the fundamental frequency and $\Gamma(t)$ is the real mass coverage of the adsorbate (macroion), which can be calculated as a function of the adsorption time from Eq. (S7).

Using this inertia load impedance as a scaling variable one obtains the following expressions connecting the normalized impedance $\overline{Z}^{*}$ components with the frequency and the dissipation shifts:[14]

$$\overline{Z}_{im} = \frac{C_s}{\Gamma(t)}[-\Delta f(t)/n_o]$$

$$\overline{Z}_{re} = -\frac{C_s}{\Gamma(t)}\overline{\Delta D}(t) \tag{S18}$$

where $C_s = \frac{Z_q}{2 f_0^2}$ is the Sauerbrey constant equal to 0.177 (mg m^{-2}) Hz^{-1} for $f_0 = 5\times10^6$ Hz.

The impedance components in the limit of low macroion coverage can be calculated using the experimental kinetic results from the following equation:[14]

$$\overline{Z}_{im} = \left(\frac{d}{dt}\frac{-\Delta f}{n_o}\right) / \left(\frac{d\Gamma}{dt}\right)$$

$$\overline{Z}_{re} = -C_s\left(\frac{d\overline{\Delta D}}{dt}\right) / \left(\frac{d\Gamma}{dt}\right) \tag{S19}$$

It is worth mentioning that Eq. (S18) is applicable for an arbitrary adsorbate shape, size and flow conditions.

These experimental data derived for PARG were interpreted in terms of the theoretical results derived previously [13] applying the *ab initio* hydrodynamic theory for spherical adsorbates (particles). In the case of the stiff contact prohibiting any particle motion relatively to the sensor, the impedance components were numerically calculated for discrete values of the $\frac{d}{2\delta}$ parameter, where d is the diameter of the particle, $\delta = \left(\frac{2\nu}{\omega}\right)^{1/2} = \left(\frac{\nu}{\pi n f_0}\right)^{1/2}$ is the hydrodynamic boundary layer thickness, is the fluid kinematic viscosity. Interestingly for $\frac{d}{2\delta}$ much smaller than unity, the hydrodynamic contribution of the normalized imaginary impedance component approached $9.64\frac{\rho}{\rho_a}$, whereas the real impedance

component increased as $-1.5\frac{d}{2\delta}$. Therefore, the normalized impedance components were explicitly given in this limit by the formula:

$$\bar{Z}_{im} = \left(1 + 9.64\frac{\rho}{\rho_a}\right)$$

$$\bar{Z}_{re} = -1.5\frac{d}{2\delta} \qquad \text{(S20)}$$

where ρ_a and ρ are the adsorbate and the fluid densities, respectively.

In the case of a soft (lubricated) contact, enabling particle sliding and rocking motions relative to the sensor, the following formula for the complex impedance normalized by the pure inertia load was derived in Ref. [15]:

$$\bar{Z} = \left(1 + \frac{3\rho}{2\rho_a}\right) i e^{-2\lambda \bar{h}} \qquad \text{(S21)}$$

where i is the imaginary unit, $\lambda = (1-i)\left(\frac{d}{2\delta}\right)$, h is the distance between the particle center and the sensor surface, $\bar{h} = 2h/d = 1 + 2h_m/d$ is the scaled distance and h_m is the minimum distance between the particle and the sensor surfaces. Notice that Eq. (S21) is only accurate for not too small minimum distance if $h_m \gtrsim d/2$ (see Ref. [15]).

For $\frac{d}{2\delta} \ll 1$, and $2h_m/d \sim 1$, Eq. (S21) can be expressed in the following form:

$$\bar{Z}_{im} = \left(1 + \frac{3\rho}{2\rho_a}\right)\left(1 - 2\frac{h}{\delta}\right)$$

$$\bar{Z}_{re} = -2\frac{h}{\delta}\left(1 + \frac{3\rho}{2\rho_a}\right)\left(1 - 2\frac{h}{\delta}\right) = -2\frac{h}{\delta}\bar{Z}_{im} \qquad \text{(S22)}$$

It should be mentioned that these equations are strictly applicable for spherically shaped adsorbates in the limit of low surface coverage. However, one can expect that they retain their basic form for anisotropic particles, with slightly different numerical coefficient appearing at the ρ/ρ_a term describing the relative significance of the hydrodynamic and inertia forces.

References

(1) Adamczyk, Z.; Sadowska, M.; Nattich-Rak, M. Quantifying Nanoparticle Layer Topography: Theoretical Modeling and Atomic Force Microscopy Investigations. *Langmuir* **2023**, *39* (42), 15067–15077. https://doi.org/10.1021/acs.langmuir.3c02024.

(2) Adamczyk, Z. Kinetics of Diffusion-Controlled Adsorption of Colloid Particles and Proteins. *J. Colloid Interface Sci.* **2000**, *229* (2), 477–489. https://doi.org/10.1006/jcis.2000.6993.

(3) Adamczyk, Z. *Particles at Interfaces: Interactions, Deposition, Structure*; Interface Science and Technology; Elsevier: Amsterdam, 2017.

(4) Hinrichsen, E. L.; Feder, J.; Jøssang, T. Geometry of Random Sequential Adsorption. *J. Stat. Phys.* **1986**, *44* (5–6), 793–827. https://doi.org/10.1007/BF01011908.

(5) Schaaf, P.; Talbot, J. Surface Exclusion Effects in Adsorption Processes. *J. Chem. Phys.* **1989**, *91* (7), 4401–4409. https://doi.org/10.1063/1.456768.

(6) Ricci, S. M.; Talbot, J.; Tarjus, G.; Viot, P. Random Sequential Adsorption of Anisotropic Particles. II. Low Coverage Kinetics. *J. Chem. Phys.* **1992**, *97* (7), 5219–5228. https://doi.org/10.1063/1.463988.

(7) Talbot, J.; Tarjus, G.; Van Tassel, P. R.; Viot, P. From Car Parking to Protein Adsorption: An Overview of Sequential Adsorption Processes. *Colloids Surf. A Physicochem. Eng. Asp.* **2000**, *165* (1), 287–324. https://doi.org/10.1016/S0927-7757(99)00409-4.

(8) Cieśla, M. Continuum Random Sequential Adsorption of Polymer on a Flat and Homogeneous Surface. *Phys. Rev. E* **2013**, *87* (5), 052401. https://doi.org/10.1103/PhysRevE.87.052401.

(9) Kosior, D.; Morga, M.; Maroni, P.; Cieśla, M.; Adamczyk, Z. Formation of Poly-l-Lysine Monolayers on Silica: Modeling and Experimental Studies. *J. Phys. Chem. C* **2020**, *124* (8), 4571–4581. https://doi.org/10.1021/acs.jpcc.9b10870.

(10) Tarnapolsky, A.; Freger, V. Modeling QCM-D Response to Deposition and Attachment of Microparticles and Living Cells. *Anal. Chem.* **2018**, *90* (23), 13960–13968. https://doi.org/10.1021/acs.analchem.8b03411.

(11) Meléndez, M.; Vázquez-Quesada, A.; Delgado-Buscalioni, R. Load Impedance of Immersed Layers on the Quartz Crystal Microbalance: A Comparison with Colloidal Suspensions of Spheres. *Langmuir* **2020**, *36* (31), 9225–9234. https://doi.org/10.1021/acs.langmuir.0c01429.

(12) Delgado-Buscalioni, R. Coverage Effects in Quartz Crystal Microbalance Measurements with Suspended and Adsorbed Nanoparticles. *Langmuir* **2024**, *40* (1), 580–593. https://doi.org/10.1021/acs.langmuir.3c02792.

(13) Leshansky, A. M.; Rubinstein, B. Y.; Fouxon, I.; Johannsmann, D.; Sadowska, M.; Adamczyk, Z. Quartz Crystal Microbalance Frequency Response to Discrete Adsorbates in Liquids. *Anal. Chem.* **2024**, *96* (26), 10559–10568. https://doi.org/10.1021/acs.analchem.4c00968.

(14) Sadowska, M.; Nattich-Rak, M.; Morga, M.; Adamczyk, Z.; Basinska, T.; Mickiewicz, D.; Gadzinowski, M. Anisotropic Particle Deposition Kinetics from Quartz Crystal Microbalance Measurements: Beyond the Sphere Paradigm. *Langmuir* **2024**, *40* (15), 7907–7919. https://doi.org/10.1021/acs.langmuir.3c03676.

(15) Fouxon, I.; Rubinstein, B. Y.; Leshansky, A. M. Excess Shear Force Exerted on an Oscillating Plate Due to a Nearby Particle. *Phys. Rev. Fluids* **2023**, *8* (5), 054104. https://doi.org/10.1103/PhysRevFluids.8.054104.
